# Supplementary material for: Comparative Gene Expression Analysis Reveals Similarities and Differences of Chronic Myeloid Leukemia Phases
Source: Cancers (Basel). 2022 Jan 5;14(1):256. doi: 10.3390/cancers14010256 (PMC8750437; doi:10.3390/cancers14010256)
Supplement: Supplementary file 1 [file cancers-14-00256-s001.zip › SupplementaryMaterials/Figure_S1.pdf]

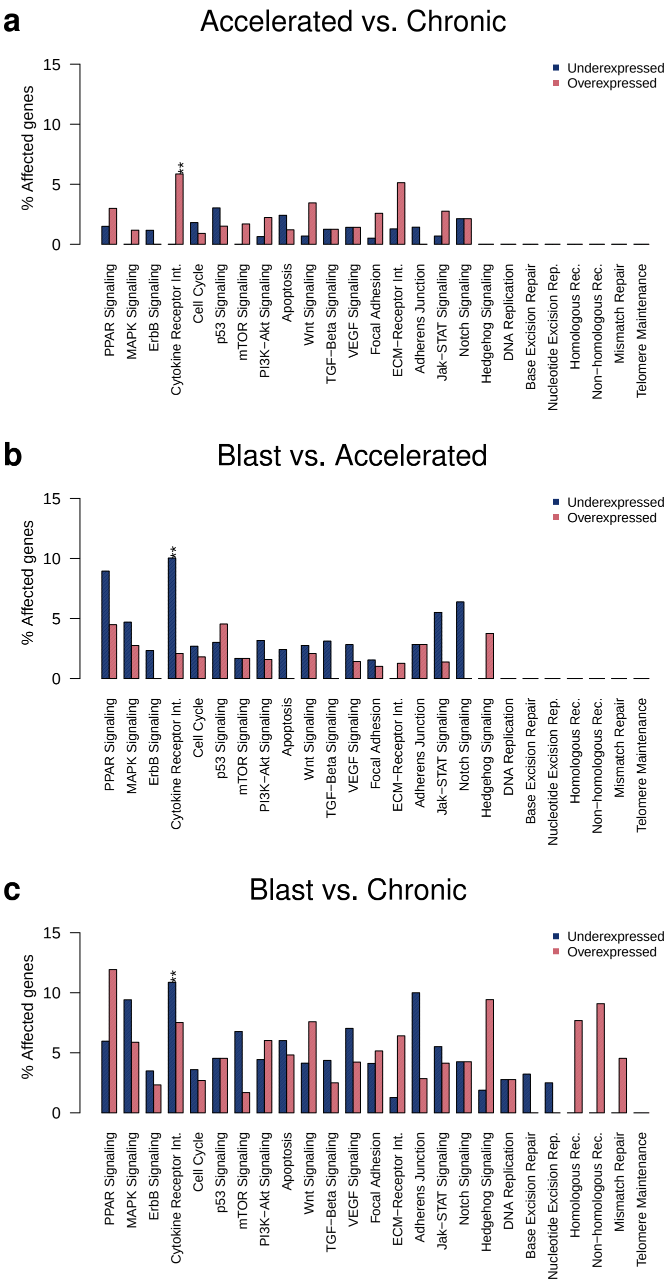

Figure S1: Gene expression alterations affecting cancer-relevant signaling pathways. Percentages of under- and overexpressed genes observed for specific signaling pathways are shown for the pairwise comparisons of the three CML phases. Differentially expressed genes at the q-value cutoff of 0.05 with an average expression change of $|\log_2\text{-ratio}| \geq 1$ were considered. Overrepresented pathways were marked by asterisks separately for an enrichment of under- or overexpressed genes (Fisher's exact test: '**' for FDR-adjuated $p < 0.01$). Signaling pathway alterations for the comparisons of accelerated to chronic phase, blast to accelerated phase, and blast to chronic phase are shown in the subpanels (**a**), (**b**), and (**c**), respectively.
