# Supplementary material for: Comparative Gene Expression Analysis Reveals Similarities and Differences of Chronic Myeloid Leukemia Phases
Source: Cancers (Basel). 2022 Jan 5;14(1):256. doi: 10.3390/cancers14010256 (PMC8750437; doi:10.3390/cancers14010256)
Supplement: Supplementary file 1 [file cancers-14-00256-s001.zip › SupplementaryMaterials/Figure_S2.pdf]

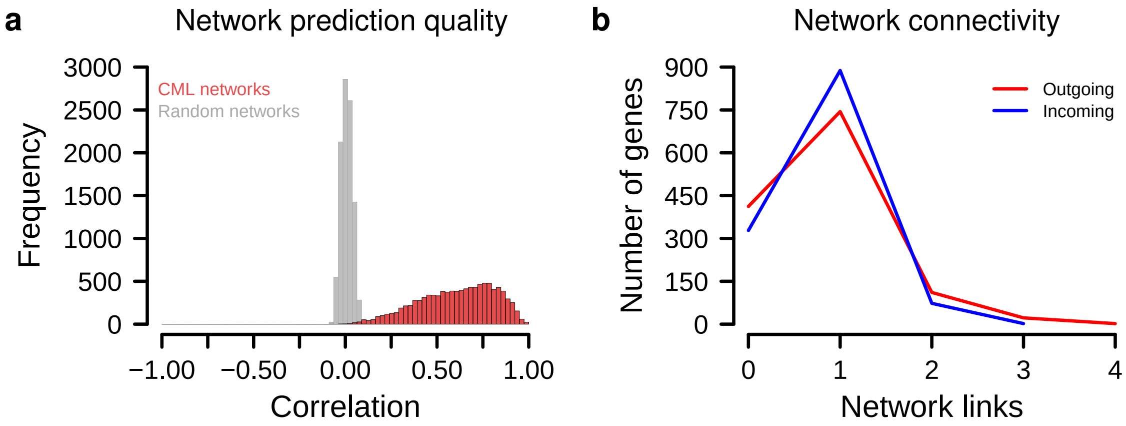

Figure S2: Prediction quality and connectivity of the CML signature-specific gene regulatory networks. (**a**) Distribution of average correlations representing the prediction quality of individual gene expression levels for the 100 learned networks (red) and their corresponding random networks of same complexity (grey). Pearson correlations between network-based predicted and originally measured expression levels of individual genes were determined for each independent network-specific test set. Only network links between genes with a q-value ≤ 0.01 were considered for the prediction of gene expression levels. (**b**) Number of genes with a specific number of outgoing and incoming network links that were present in at least 90 of 100 networks for a network link q-value cutoff of 0.01.
