# Supplementary material for: Comparative Gene Expression Analysis Reveals Similarities and Differences of Chronic Myeloid Leukemia Phases
Source: Cancers (Basel). 2022 Jan 5;14(1):256. doi: 10.3390/cancers14010256 (PMC8750437; doi:10.3390/cancers14010256)
Supplement: Supplementary file 1 [file cancers-14-00256-s001.zip › SupplementaryMaterials/Text_S1.pdf]

Comparative gene expression analysis reveals similarities and differences of chronic myeloid leukemia phases

Text S1: Literature analysis of potential major regulators that differed in their expression between CML phases

We learned CML-specific gene regulatory networks that were associated with the observed expression differences between the three CML phases. These networks also included 24 genes with phase-specific expression behavior that had an increased connectivity to other differentially expressed genes (see Table 1 in main manuscript). We performed an in-depth literature analysis to determine known cellular functions of these genes. These functions in combination with the characteristic phase-specific gene expression patterns can contribute to a better characterization of differences between the three CML phases.

- We found that NDUFAB1, SPRR2A and AURKB could contribute to the accumulation of additional mutations. NDUFAB1 encodes a subunit of an oxidoreductase that is part of the respiratory chain, whose enhanced activity in CML can lead to increased levels of reactive oxygen species that can damage DNA [Flis et al., 2012]. SPRR2A protects cells from oxidative stress in benign and malignant liver disease [Mizuguchi et al., 2014]. AURKB is involved in chromatid segregation [Bose et al., 2019]. The observed significant upregulation of NDUFAB1 in the blast crisis, the significant downregulation of SPRR2A in the accelerated phase and the blast crisis, and the significant downregulation of AURKB in the blast crisis are in accordance with the accumulation of additional mutations in CML progression [Nowicki et al., 2004, Cramer et al., 2008, Rabian et al., 2019].

- We found that HLA-B, HLA-DMB, HLA-DRA, MUC8 and OPTN are involved in the regulation of immune responses and inflammation. Increased cytotoxic T lymphocyte responses have been reported for a negative association between the HLA-B antigen expression and the presence of the BCR-ABL fusion in leukemia patients [de Carvalho et al., 2012]. Overexpression of HLA-DMB has been reported for CML [Kremer et al., 2014]. MUC8 has been associated with anti-inflammatory processes [Cha and Song, 2018]. The observed upregulation of HLA-DMB, HLA-DRA and OPTN and the downregulation of HLA-B and MUC8 in the blast crisis could indicate a role of the immune system in CML progression.

- We found that several genes could also support the observed global deregulation of cancer-relevant signaling pathways. Reduced CDCA3 expression has led to decreased cell proliferation in acute myeloid leukemia [Bi et al., 2018]. Downregulation of EN1 has been reported to increase activity of WNT-, Notch- and Hedgehog signaling [Peluffo et al., 2019]. A targeted inhibition of RPL18A expression has led to a lack of mature red blood cells via the activation of p53 and Jak-Stat signaling in a zebrafish model [Chen et al., 2020]. ADD2 is involved in the regulation of cell proliferation and migration [Luo and Shen, 2017, Yang et al., 2018]. TMEM40 is involved in the regulation of apoptosis and cell proliferation via alterations of p53 signaling [Zhang et al., 2018]. Downregulation of TMEM40 has led to reduced cell proliferation, migration and invasion in different types of

cancer [Zhang et al., 2018, Zhang et al., 2019, Liu et al., 2020]. Overexpression of CEACAM6 has been reported to be involved in the regulation of cell adhesion, proliferation, differentiation, apoptosis, invasion and metastasis formation [Rizeq et al., 2018]. Therefore, the observed downregulation of ADD2, CDCA3, EN1 and CEACAM6 in the blast crisis, the upregulation of TMEM40 in the accelerated phase and the blast crisis, and the upregulation of RPL18A in the blast crisis could also contribute to the observed increase of differential expression of cancer-relevant signaling pathways in CML progression.

- We found that some of the genes have also been associated with survival or treatment response in leukemia and other types of cancer. The chemokine PF4 (also known as CXCL4) is involved in the regulation of hematopoietic stem and progenitor cells [Bruns et al., 2014, Sinclair et al., 2016]. A recovery of PF4 to the normal protein level has been associated with a complete remission of acute myeloid leukemia [Kim et al., 2008, Bai et al., 2013] and increased PF4 expression has been associated with better therapy response and improved survival of CML patients in blast crisis [Ryo et al., 1991]. The chemokine AZU1 has also been associated with CML therapy response and survival [Yong et al., 2006, Cha et al., 2016]. CDCA3 has been reported to influence cisplatin sensitivity in lung cancer [O'Byrne et al., 2016]. EN1 overexpression has been associated with resistance of breast cancer to chemotherapy [Beltran et al., 2014]. Thus, the observed upregulation of PF4 in the accelerated and blast phase, the downregulation of CDCA3 and EN1 in the blast phase, and the phase-wise reduction of the AZU1 expression from the chronic to the blast phase could also have an impact on treatment responses and potentially contribute to the observation that targeted treatments are less efficient in advanced CML phases [Palandri et al., 2008, Silver et al., 2009, Houshmand et al., 2019].
- We found that PRG3 and TLX3 have been identified as important driver genes in other leukemias. PRG3 encodes a proteoglykane that has been reported as major regulator associated with survival of acute myeloid leukemia patients [Tan et al., 2020]. TLX3 encodes a homeobox transcription factor that is known as a master regulator of T cell acute lymphoblastic leukemia [Gatta et al., 2012].

References

[Bai et al., 2013] Bai, J., He, A., Zhang, W., Huang, C., Yang, J., Yang, Y., Wang, J., and Zhang, Y. (2013). Potential biomarkers for adult acute myeloid leukemia minimal residual disease assessment searched by serum peptidome profiling. *Proteome Sci*, 11:39.

[Beltran et al., 2014] Beltran, A. S., Graves, L. M., and Blancafort, P. (2014). Novel role of Engrailed 1 as a prosurvival transcription factor in basal-like breast cancer and engineering of interference peptides block its oncogenic function. *Oncogene*, 33(39):4767–77.

[Bi et al., 2018] Bi, L., Zhou, B., Li, H., He, L., Wang, C., Wang, Z., Zhu, L., Chen, M., and Gao, S. (2018). A novel miR-375-HOXB3-CDCA3/DNMT3B regulatory circuitry contributes to leukemogenesis in acute myeloid leukemia. *BMC Cancer*, 18(1):182.

[Bose et al., 2019] Bose, A., Sudevan, S., Rao, V. J., Shima, H., Trivedi, A. K., Igarashi, K., and Kundu, T. K. (2019). Haploinsufficient tumor suppressor Tip60 negatively regulates oncogenic Aurora B kinase. *J Biosci*, 44(6):147.

[Bruns et al., 2014] Bruns, I., Lucas, D., Pinho, S., Ahmed, J., Lambert, M. P., Kunisaki, Y., Scheiermann, C., Schiff, L., Poncz, M., Bergman, A., and Frenette, P. S. (2014). Megakaryocytes regulate hematopoietic stem cell quiescence through CXCL4 secretion. *Nat Med*, 20(11):1315–20.

[Cha and Song, 2018] Cha, H.-J. and Song, K. S. (2018). Effect of MUC8 on Airway Inflammation: A Friend or a Foe? *J Clin Med*, 7(2):26.

[Cha et al., 2016] Cha, K., Li, Y., and Yi, G.-S. (2016). Discovering gene expression signatures responding to tyrosine kinase inhibitor treatment in chronic myeloid leukemia. *BMC Med Genomics*, 9(Suppl 1):29.

[Chen et al., 2020] Chen, C., Lu, M., Lin, S., and Qin, W. (2020). The nuclear gene rpl18 regulates erythroid maturation via JAK2-STAT3 signaling in zebrafish model of Diamond–Blackfan anemia. *Cell Death Dis*, 11(2):135.

[Cramer et al., 2008] Cramer, K., Nieborowska-Skorska, M., Koptyra, M., Slupianek, A., Penserga, E. T. P., Eaves, C. J., Aulitzky, W., and Skorski, T. (2008). BCR/ABL oncogenic kinase promotes unfaithful repair of the reactive oxygen species–dependent DNA double-strand breaks. *Cancer Res*, 68(17):6884–8.

[de Carvalho et al., 2012] de Carvalho, D. L., Barbosa, C. D., de Carvalho, A. L., and Beck, S. T. (2012). Association of HLA antigens and BCR-ABL transcripts in leukemia patients with the Philadelphia chromosome. *Rev Bras Hematol Hemoter*, 34(4):280–284.

[Flis et al., 2012] Flis, K., Irvine, D., Copland, M., Bhatia, R., and Skorski, T. (2012). Chronic myeloid leukemia stem cells display alterations in expression of genes involved in oxidative phosphorylation. *Leuk Lymphoma*, 53(12):2474–8.

[Gatta et al., 2012] Gatta, G. D., Palomero, T., Perez-Garcia, A., Ambesi-Impiombato, A., Bansal, M., Carpenter, Z. W., De Keersmaecker, K., Sole, X., Xu, L., Paietta, E., Racevskis, J., Wiernik, P. H., Rowe, J. M., Meijerink, J. P., Califano, A., and Ferrando, A. A. (2012). Reverse engineering of TLX oncogenic transcriptional networks identifies RUNX1 as tumor suppressor in T-ALL. *Nat Med*, 18(3):436–40.

[Houshmand et al., 2019] Houshmand, M., Simonetti, G., Circosta, P., Gaidano, V., Cignetti, A., Martinelli, G., Saglio, G., and Gale, R. P. (2019). Chronic myeloid leukemia stem cells. *Leukemia*, 33(7):1543–1556.

[Kim et al., 2008] Kim, J. Y., Song, H.-J., Lim, H.-J., Shin, M.-G., Kim, J. S., Kim, H.-J., Kim, B. Y., and Lee, S. (2008). Platelet factor-4 is an indicator of blood count recovery in acute myeloid leukemia patients in complete remission. *Mol Cell Proteomics*, 7(2):431–41.

[Kremer et al., 2014] Kremer, A. N., van der Meijden, E. D., Honders, M. W., Pont, M. J., Goeman, J. J., Falkenburg, J. H. F., and Griffioen, M. (2014). Human leukocyte antigen-DO regulates surface presentation of human leukocyte antigen class II-restricted antigens on B cell malignancies. *Biol Blood Marrow Transplant*, 20(5):742–7.

[Liu et al., 2020] Liu, D., Zhou, G., Shi, H., Chen, B., Sun, X., and Zhang, X. (2020). Down-regulation of Transmembrane protein 40 by miR-138-5p Suppresses Cell Proliferation and Mobility in Clear Cell Renal Cell Carcinoma. *Iran J Biotechnol*, 18(1):e2270.

[Luo and Shen, 2017] Luo, C. and Shen, J. (2017). Adducin in tumorigenesis and metastasis. *Oncotarget*, 8(29):48453–48459.

[Mizuguchi et al., 2014] Mizuguchi, Y., Isse, K., Specht, S., Lunz, J. G., Corbitt, N., Takizawa, T., and Demetris, A. J. (2014). Small proline rich protein 2a in benign and malignant liver disease. *Hepatology*, 59(3):1130–1143.

[Nowicki et al., 2004] Nowicki, M. O., Falinski, R., Koptyra, M., Slupianek, A., Stoklosa, T., Gloc, E., Nieborowska-Skorska, M., Blasiak, J., and Skorski, T. (2004). BCR/ABL oncogenic kinase promotes unfaithful repair of the reactive oxygen species–dependent DNA double-strand breaks. *Blood*, 104(12):3746–3753.

[O'Byrne et al., 2016] O'Byrne, K., Adams, M., Burgess, J., and Richard, D. (2016). CDCA3 regulates the cell cycle and modulates cisplatin sensitivity in non-small cell lung cancer. *Journal of Thoracic Oncology*, 11(4):S65.

[Palandri et al., 2008] Palandri, F., Castagnetti, F., Testoni, N., Luatti, S., Marzocchi, G., Bassi, S., Breccia, M., Alimena, G., Pungolino, E., Rege-Cambrin, G., Varaldo, R., Miglino, M., Specchia, G., Zuffa, E., Ferrara, F., Bocchia, M., Saglio, G., Pane, F., Alberti, D., Martinelli, G., Baccarani, M., Rosti, G., and GIMEMA Working Party on Chronic Myeloid Leukemia (2008). Chronic myeloid leukemia in blast crisis treated with imatinib 600 mg: outcome of the patients alive after a 6-year follow-up. *Haematologica*, 93(12):1792–6.

[Peluffo et al., 2019] Peluffo, G., Subedee, A., Harper, N. W., Kingston, N., Jovanovic, B., Flores, F., Stevens, L. E., Beca, F., Trinh, A., Chilamakuri, C. S. R., Papachristou, E. K., Murphy, K., Su, Y., Marusyk, A., D'Santos, C. S., Rueda, O. M., Beck, A. H., Caldas, C., Carroll, J. S., and Polyak, K. (2019). En1 is a transcriptional dependency in triple-negative breast cancer associated with brain metastasis. *Cancer Res*, 79(16):4173–4183.

[Rabian et al., 2019] Rabian, F., Lengline, E., and Rea, D. (2019). Towards a Personalized Treatment of Patients with Chronic Myeloid Leukemia. *Current Hematologic Malignancy Reports*, 14:492–500.

[Rizeq et al., 2018] Rizeq, B., Zakaria, Z., and Ouhtit, A. (2018). Towards understanding the mechanisms of actions of carcinoembryonic antigen-related cell adhesion molecule 6 in cancer progression. *Cancer Sci*, 109(1):33–42.

[Ryo et al., 1991] Ryo, R., Adachi, M., Sugano, W., Yasunaga, M., Yoshida, A., Jikai, J., Saigo, K., Yamaguchi, N., Akita, H., Yokoyama, M., Konaka, Y., and Poncz, M. (1991). Platelet factor 4 mrna expression in cells from a patient with megakaryoblastic crisis of chronic myelogenous leukemia. *Cancer*, 67(4):960–4.

[Silver et al., 2009] Silver, R. T., Cortes, J., Waltzman, R., Mone, M., and Kantarjian, H. (2009). Sustained durability of responses and improved progression-free and overall survival with imatinib treatment for accelerated phase and blast crisis chronic myeloid leukemia: long-term follow-up of the STI571 0102 and 0109 trials. *Haematologica*, 94(5):743–4.

[Sinclair et al., 2016] Sinclair, A., Park, L., Shah, M., Drotar, M., Calaminus, S., Hopcroft, L. E. M., Kinstrie, R., Guitart, A. V., Dunn, K., Abraham, S. A., Sansom, O., Michie, A. M., Machesky, L., Kranc, K. R., Graham, G. J., Pellicano, F., and Holyoake, T. L. (2016). CXCR2 and CXCL4 regulate survival and self-renewal of hematopoietic stem/progenitor cells. *Blood*, 128(3):371–83.

[Tan et al., 2020] Tan, Y., Zheng, L., Du, Y., Zhong, Q., Zhu, Y., Liu, Z., Liu, S., and Zhang, Q. (2020). Identification of the hub genes and pathways involved in acute myeloid leukemia using bioinformatics analysis. *Medicine (Baltimore)*, 99(35):e22047.

[Yang et al., 2018] Yang, C., Sui, Z., Xu, T., Liu, W., Wang, X., and Zeng, X. (2018). Lipid raft-associated β-adducin participates in neutrophil migration. *Mol Med Rep*, 18(2):1353–1360.

[Yong et al., 2006] Yong, A. S. M., Szydlo, R. M., Goldman, J. M., Apperley, J. F., and Melo, J. V. (2006). Molecular profiling of CD34+ cells identifies low expression of CD7, along with high expression of proteinase 3 or elastase, as predictors of longer survival in patients with CML. *Blood*, 107(1):205–212.

[Zhang et al., 2019] Zhang, Q., Huang, D., Zhang, Z., Feng, Y., Fu, M., Wei, M., Zhou, J., Huang, Y., Liu, S., and Shi, R. (2019). High expression of TMEM40 contributes to progressive features of tongue squamous cell carcinoma. *Oncol Rep*, 41(1):154–164.

[Zhang et al., 2018] Zhang, Z.-F., Zhang, H.-R., Zhang, Q.-Y., Lai, S.-Y., Feng, Y.-Z., Zhou, Y., Zheng, S.-R., Shi, R., and Zhou, J.-Y. (2018). High expression of TMEM40 is associated with the malignant behavior and tumorigenesis in bladder cancer. *J Transl Med*, 16(1):9.
