# Supplementary material for: Comparative Gene Expression Analysis Reveals Similarities and Differences of Chronic Myeloid Leukemia Phases
Source: Cancers (Basel). 2022 Jan 5;14(1):256. doi: 10.3390/cancers14010256 (PMC8750437; doi:10.3390/cancers14010256)
Supplement: Supplementary file 1 [file cancers-14-00256-s001.zip › SupplementaryMaterials/Figure_S3.pdf]

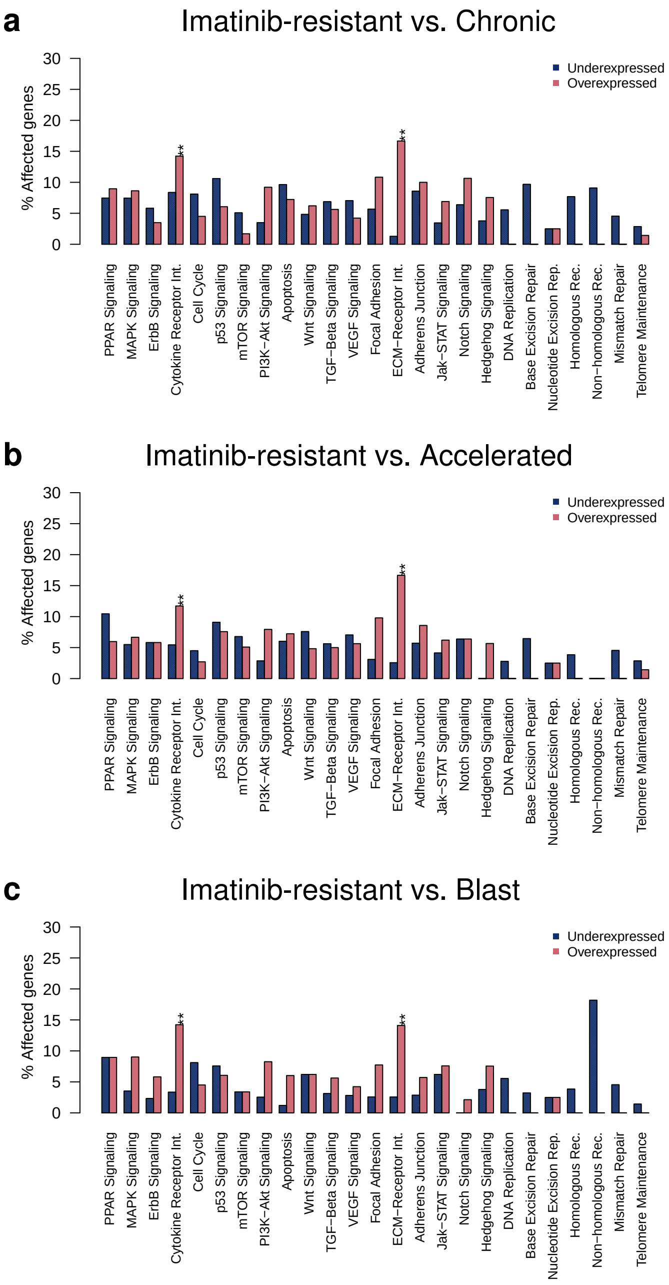

Figure S3: Signaling pathway alterations distinguishing Imatinib-resistant patients from patients of the three CML phases. Percentages of under- and overexpressed genes observed for specific signaling pathways are shown for each pairwise comparison. Differentially expressed genes at the q-value cutoff of 0.05 with an average absolute expression change of $|\log_2\text{-ratio}| \geq 1$ were considered. Overrepresented pathways were marked by asterisks separately for an enrichment of under- or overexpressed genes (Fisher's exact test: '**' for FDR-adjusted $p < 0.01$). Individual signaling pathway alterations for the comparison of Imatinib-resistant CML patients to the patients of the three CML phases are shown in the subpanels (**a**), (**b**), and (**c**).
